# On Malaria Transmission and Transmission Blocking Immunity

**DOI:** 10.4269/ajtmh.21-1319

**Published:** 2022-07-11

**Authors:** Richard Carter, Louis H. Miller, Richard Culleton

**Affiliations:** ^1^ National Institute of Allergy and Infectious Diseases, Bethesda, Maryland;; ^2^ Division of Molecular Parasitology, Proteo-Science Center, Ehime University, Japan

*Editors’ Note: The following manuscript is an abbreviated version of a volume of memoirs written by Richard Carter from 2015 to 2018. The unabridged version, *Studies with Malaria: A Memoir—Part 2: On Malaria Transmission and Transmission Blocking Immunity,* is currently housed at the Wellcome Collection, London.*^1^* We have attempted to distill the essence of the original volume into a single manuscript. This has necessarily involved the removal of large sections of text dealing with several important developments involving multiple notable researchers. We apologize for these omissions.*

*In their original form, Richard’s memoirs were written as a series of e-mails to a student in response to questions regarding the origin of particular topics in malariology. As such, they are written informally and in a rather esoteric style, which we have sought to preserve—idiosyncrasies and all. We urge interested readers to seek out the unabridged version at the Wellcome Collection or by contacting either of us.*

*—Louis H. Miller and Richard Culleton, Guest Editors*

Following my introduction to a life in science in the *Genetics Department* at the *University of Edinburgh,* I moved in the Fall of 1974 to the *National Institutes of Health* (NIH) in Bethesda, Maryland. In Edinburgh, I had worked in the laboratory of Professor Geoffrey Beale, first for my PhD on aspects of the genetics of malaria parasites, and for three years following as a Research Assistant on the same. With the mentorship of Professor P.C.C. Garnham of the *London School of Hygiene and Tropical Medicine*, who, with Professor Beale, was the co-originator of the malaria genetics project in Edinburgh, I had been accepted for a two-year postdoctoral appointment in the laboratory of Dr Louis H. Miller, the newly appointed head of the *Malaria Section*, in the *National Institute of Allergy and Infectious Diseases* (NIAID), of the NIH.

I came to the NIH with the idea of trying to conduct a genetic cross between two ‘strains’ of the human malaria parasite, *Plasmodium falciparum*. Within the first few days of my arrival, discussions on my idea were held with Lou Miller and with his deputy head of Section, Dr Robert (Bob) Gwadz, an entomologist (Lou himself was a medical doctor who had come into contact with, and become interested in, malaria in Vietnam). As always, finding the right biological system with which to attempt a genetic cross between two malaria parasites was the key.

The immediate problem was the infectivity of *P. falciparum*- or *P. vivax*-infected *Aotus* monkeys to mosquitoes. There seemed to be no reliable pattern of gametocyte production during such infections and consequently no predictable pattern of infectivity of the parasites to mosquitoes. Without a reliable system of infectivity, there were no prospects for attempting a genetic cross.

One possible way around this, I thought, could be to attempt to freeze gametocytes in viable form from each of the projected strains of parasite to be used in a cross, as and when they might appear during infections in *Aotus* monkeys.

It was agreed that I should start by trying to achieve this objective – I mean viably freezing infectious gametocytes. However, to practice for attempting this with infections in *Aotus* monkeys, I was to experiment with the far less expensive and thoroughly more amenable chicken malaria parasite, *Plasmodium gallinaceum*, and its laboratory vector mosquito, *Aedes aegypti*. This system, in all its components, was maintained in the *Laboratory of Parasitic Diseases*, and I set out, forthwith, upon the study of gametocytes of *P. gallinaceum* in chickens.

I ran immediately into the problem that anyone who has worked with live malarial gametocytes discovers. Soon after drawing gametocyte-infected blood from its host, be it a bird, a mouse, a monkey or a human, there is a strong tendency for the gametocytes to undergo an irreversible transformation. It is the transformation from intracellular sexual parasites within the confines of a host red blood cell to extracellular male and female gametes. In the case of the male gametes, these are vigorously active flagellated forms wriggling through the blood plasma, or whatever physiological solution they may have been collected into.

I was suggested to read the papers by Anne Bishop.^2^^,^^3^ I did, and they were fascinating. Instead of asking what might stop gametocytes from exflagellating, she had asked what is it in chicken blood that causes gametocytes to exflagellate. She had done her work in Cambridge (England) in the 1950s. In a very extensive series of experiments, trying one combination of salts that are present in chicken plasma after another, she concluded that the only one whose presence was essential for gametocytes to be able to exflagellate is the *bicarbonate ion*.^2^ Actually, to maintain the correct ‘osmotic pressure’ of the solution – without which red blood cells and their contents, e.g., malaria parasites, burst open – the bicarbonate was always dissolved in a ‘physiological’ saline solution consisting of the natural (physiological) concentration of sodium chloride in chicken plasma and a ‘buffer’ to keep a physiological pH (acidity). The ‘buffer’ was a substance (artificial because it is not in chicken or any other blood) called Tris. So that was that. Bicarbonate ion in a physiological saline was all you needed in a solution in which gametocytes are suspended in order for them to be able to ‘exflagellate’. But I wanted to know: what do you do to stop them from exflagellating? It was actually a no-brainer, but it was not me but Louis Miller, typically, who pointed it out; remove bicarbonate.

And that is what I did. And, of course, it worked. Wash gametocyte-infected blood immediately in a suitably large volume of Tris-buffered sodium chloride physiological saline – without bicarbonate in it – and after half an hour they will never exflagellate . . . never, whatever you do . . . I mean never, ever again.

And then the obvious, almost, occurred to me. The poor little things may be starving to death. They may need some glucose to keep going. And they do indeed. With a nice physiological concentration of glucose added to the Tris-buffered saline, I could hold the gametocytes perfectly happy, and induce exflagellation any time I liked just by returning the gametocytes to the bicarbonate-containing saline. Indeed, I found that I could easily hold the gametocytes in the glucose-containing Tris-buffered saline all day long, and all night, if need be, and stimulate them to undiminished exflagellation at the end of it.

I was so pleased. I was delighted, actually. It was a perfect game. Like Eeyore in Winnie-the-Pooh, I could put my burst balloon (though mine was not burst, that is the whole point) into my jar and I could take it out again, just as good as when it went in. I could hold gametocytes in suspension, literally in the palm of my hand, and I could turn them on to exflagellate at will. It was magic. Indeed, I felt like a magician. I was tempted to call this magical solution ‘*Happy Medium*’. I resisted. But, still in my youthful enthusiasm, I named it (the Tris-buffered, glucose-containing, sodium chloride physiological saline – without bicarbonate) *Suspended Animation* solution. My mind turned to the question that Anne Bishop had asked: what does cause gametocytes to exflagellate?

The problem Mary Nijhout, a new postdoctoral fellow, and I faced was how to independently manage and measure the three mutually interactive variables that we believed were involved – bicarbonate concentration, CO_2_ partial pressure (as is the technically correct way of putting and thinking about this) and pH. In short order, Mary identified and put together from within the magic kingdom of many possibilities that was the NIH, two special pieces of equipment that exactly met the needs of these experiments.

One of these was an exquisite piece of engineering called a ‘*Dvorak-Stotler Chamber*’. It was the invention of Dr James Dvorak who was a technological genius. His ‘*Chamber*’ allowed living cells to be perfused with any solution under any reasonable gas tension that you liked while watching their behavior, e.g., merozoites invading red blood cells, or gametocytes exflagellating, through a high-power light microscope and, if desired, photographing them or recording their behavior with a video camera.

The second piece of equipment was a ‘*blood gas analyser*’, and what it did was measure the partial pressures of oxygen and carbon dioxide (CO_2_), and also the pH, in whatever solution was introduced into it. I won’t try to continue with technical details but by putting the Dvorak Chamber together with the blood gas analyser, Mary was able to observe the behavior of gametocytes in any solution that we exposed them to while simultaneously being able to measure CO_2_ and pH. Bicarbonate ion concentration we took to be whatever we had prepared it to be. The essence of the experiment was to observe the amount of exflagellation by gametocytes exposed to all practically possible bicarbonate and CO_2_ concentrations and pHs. We then analyzed the results to see which of these variables were associated with the amount of exflagellation.

The answer was striking and ‘beautiful’, as an experimental scientist thinks of beauty, i.e., clear cut, unequivocal and, in its own terms, informative.^4^^,^^5^ Within the range of concentration and partial pressure we were able to apply, neither bicarbonate nor CO_2_ had any direct effect upon the amount of exflagellation that was induced. The pH, on the other hand, determined it exactly. Below pH 7.8 and above pH 8.6, there was absolutely no exflagellation at all. However, Anne Bishop showed that exflagellation occurred in the mosquito at pH 7.7. For Mary, this was not the end of the game. Indeed, it was just the beginning. And perhaps not even that, because her next step simply walked right off the page. Instead of agonizing over whether mosquito blood meals did or did not achieve the pH necessary to trigger exflagellation, according to the results we had found, she asked another question altogether. It was in fact: ‘*What happens Nature’s way*’?

Mary took gametocyte-infected blood, thoroughly washed in *Suspended Animation* medium (just saline and glucose with Tris buffer at pH7.4 and no bicarbonate) and fed it – the gametocyte-infected blood cells, in that bicarbonate-free Suspended Animation medium – to mosquitoes through a membrane feeder. It meant that the gametocytes were now inside the mosquito midgut, entirely without the bicarbonate that Anne Bishop’s experiments had shown, as ours had confirmed, is essential of gametogenesis in vitro.

After they had been in this bicarbonate-free medium inside the mosquitoes for about 10 minutes, Mary examined their stomach contents under a microscope. To my personal total astonishment, the gametocytes were exflagellating – like crazy! To cut the story short, Mary went on to demonstrate conclusively that the mosquitoes had in their stomachs a substance that potently stimulates malarial gametocytes to undergo gametogenesis (exflagellation) without any involvement of the bicarbonate-dependent, pH-controlled mechanism we had just so beautifully demonstrated. It was a lesson to me in making assumptions in science, or I suppose in any context, that I never forgot and often ponder.

Mary was able to show that the substance is present in some, but not all, other mosquito tissues than the midgut. Most potently, it is present in the head of the insect (male or female – interesting, as males don’t take blood meals, let alone transmit malaria). The active factor could be recovered in relatively clean form by feeding mosquitoes with Suspended Animation solution and harvesting the drops of liquid that some species express from the rear during feeding. She called the material (then, as yet unidentified) *Mosquito Exflagellation Factor* (MEF).^6^

As years and decades rolled by, attempts were made in various laboratories to try to discover exactly what MEF is. Success, or lack of it, was, or so I have long imagined, down to the technological power of instrumentation – of something called mass spectrometry. Then, apparently independently, two groups succeeded, reporting their finding (it was the exact same molecule) in 1998. One was that of Ron Rosenberg at the *Walter Reed Army Institute of Research* (WRAIR).^7^^,^^8^ Together with his colleagues, Rosenberg had come within a whisker of identifying MEF, in the previous year and, in reporting their result, renamed the active entity ‘*Gamete Activation Factor*’ (GAF).^7^ Why did they do this . . . I mean rename it? I don’t get it. Anything could be ‘*Gamete Activation Factor*’, out of a tin, extracted from a mushroom . . . anything. *Mosquito Exflagellation Factor* (MEF) tells you not only what it does, but where it comes from . . . a mosquito. As far as I am concerned it is, and always will be, MEF. The other success was at the hands of Oliver Bilker and colleagues in the laboratories of Professors R.E. (Bob) Sinden (malariologist) and H.R. Morris (biochemist and mass spectrometrist) at *Imperial College* in London.^9^ The answer was *xanthurenic acid.*

Malaria parasites, or their ancestors, evolved a relationship with mosquitoes and it involved picking up upon an infallible cue that you, a gametocyte, are no longer inside – for example, a chicken – but are now in a mosquito midgut. And what might be that infallible signal that you are in a mosquito midgut? No, not some feeble rise in the pH of your environment. It is the presence of xanthurenic acid.^7^^–^^9^ And, Mary by the way, had showed, pH is irrelevant within any reasonable physiological range, with or without bicarbonate ion, to the stimulation of exflagellation by MEF/XA.

## I BEGIN TO THINK ABOUT VACCINATION TO BLOCK THE TRANSMISSION OF MALARIA

This was the period of vaccine development in malaria with Ruth Nussenzweig working on sporozoite immunity in *P. berghei* and Sydney Cohen working on blood stage immunity with *P. knowlesi* merozoites. Both played their role in the discovery of vaccines many years later, but transmission blocking immunity started with myself and Bob Gwadz at NIH. But before that, among a variety of things that Clay Huff had investigated concerning malaria infectivity to mosquitoes, had been host immunity. He had found that turkeys pre-immunized with formalin-killed *Plasmodium fallax* gametocyte-containing infected blood had reduced subsequent infectivity of *P. fallax* blood infections to mosquitoes. Likewise, immunizations with formalin-fixed *P. gallinaceum*-infected blood in chickens had reduced subsequent infectivity to mosquitoes by around 90%.^10^

The first hints that anything of the sort might occur had come, however, from the work of Don Eyles, right here in the Laboratory of Parasitic Diseases itself. He had demonstrated that there were factors in the blood of infected birds that reduced the infectivity of *P. gallinaceum* to chickens.^11^ Vaccines against an infectious disease have actually two distinct, but normally simultaneously attempted, objectives. One is to protect each individual who is vaccinated against the disease; the other is to prevent and ultimately eliminate the spread of the disease. Indeed, I took it for granted in my mind, that this second objective, ‘blocking transmission’, is actually the primary purpose of vaccination. The same would, of course, apply to malaria, I thought. I would soon find that in malaria, the conventional thinking was the exact opposite; indeed, for a long time ‘transmission blocking immunity’, ‘vaccinating mosquitoes’, ‘the altruistic vaccine’ would be a laughingstock amongst all but a minority of malaria researchers. But I had not the remotest suspicion that this could be the case as I thought my thoughts that day.

Now in malaria, unlike, say, polio or measles or smallpox or, indeed, any disease for which there was a vaccine at the time, the stages which cause the disease – vaccine objective number one, above – namely, the asexual blood stage parasites, are totally different from the stages which mediate the transmission of the disease –vaccine objective number two, above – namely, the sexual stages of the parasites.

Because the sexual stage malaria parasites, the gametocytes, are the products of the asexual blood stage parasites, you might think that a vaccine that killed a large proportion of the asexual blood stage parasites would likewise reduce the infectivity of a vaccinated person to mosquitoes. I was pretty certain that killing large numbers of asexual parasites – and thereby reducing the numbers of the sexual stage parasites – would have minimal effect on the amount of malaria transmission. And the reason for this is that I already knew, from years of work in Edinburgh, that the probability of infecting a mosquito has a very poor relationship with the densities of gametocytes in the blood.

Extraordinarily low densities of gametocytes can be as infectious to mosquitoes as high densities of gametocytes. *Ipso facto*, reducing the densities of asexual parasites, e.g., with an anti-asexual parasite vaccine, and, in consequence, reducing the densities of gametocytes in the blood circulation, would have a negligible effect on infectivity to mosquitoes. I was sure that to reduce infectivity of malarial infections to mosquitoes, it would be necessary to make a vaccine that directly prevented gametocytes from infecting mosquitoes.

Meanwhile, back in early 1975, in all the talk of immunity and gametocytes, there was no comment or discussion upon how it would happen. I suppose there was nothing to talk about. Like all other immunity, it would happen in the blood circulation, or somewhere in the body. Within minutes of having this thought, the desire to do the experiment to test it was growing powerfully. But it was not my area. It – thinking about immunity and infectivity to mosquitoes – was Bob Gwadz’s. And yet, ‘making gametes’, i.e., obtaining relatively pure preparations of these stages of malaria parasites, was my thing and something that was possible only through my work. Bob Gwadz worked with whole infected blood containing gametocytes. He was not able, or in a position, to make malaria gametes unless he did so through my expertise, and he had never spoken of doing so for any purpose.

My suggestion was to test the effect of immunizing chickens with extracellular gametes of *P. gallinaceum* as a means of inducing immunity that would suppress the infectivity of a subsequently induced blood infection of *P. gallinaceum* in these same chickens to mosquitoes. The point of the immunity was that it would be against the extracellular gametes themselves and take effect after, and not before, they had emerged from gametocytes; and this would take place inside the stomach of a blood-feeding mosquito. Bob’s stated objective was to investigate whether factors induced during natural blood infections of malaria affected their infectivity to mosquitoes. There was no direct conflict between this and my proposal to induce immunity artificially by immunization with gametes.

## AND SO, IT BEGINS

On my return to the NIH from a brief summer visit back to the UK, I was more than a little taken aback to find that Bob, using the *P. gallinaceum* chicken system, had resuscitated Clay Huff’s 1950s protocol^10^ (of which I was still, at that time, unaware) of immunizing with formalin-killed gametocyte-containing blood, and had induced an immunity in chickens that greatly suppressed the infectivity of the parasites to *Aedes aegyti* mosquitoes, our laboratory vector for *P. gallinaceum*. Using the membrane feeding method, he was able to show that the effect was entirely mediated by the plasma of the immune birds and that it acted against the parasites – not while they were in the blood, but only once they were inside the mosquito midgut following the blood meal. Finally, when he had taken serum from the effectively immunized birds and added it to *P. gallinaceum* gametocyte-containing blood cells that were allowed to exflagellate (as they do spontaneously in a drop on a microscope slide – it’s the fall in CO_2_ partial pressure on exposure to air, bicarbonate-dependent, pH-controlled mechanism that Mary and I had clarified), he witnessed the male gametes rapidly agglutinate.^12^

It was game, set and match, exactly (almost, but see below) as I had imagined. ‘Ideas are cheap’, Bob told me when I suggested that it was a little derivative of what I had expounded to him before the summer.

Given the mood in the world of malaria at the time, it is unlikely that Bob did not all along have in mind the idea of testing some kind of gametocyte-related, anti-infectivity vaccine. When I put forward my proposal to immunize with gametes, it would have jump-started him to do something along those lines, assuming, that is, that he had not already planned to do so at the time that I spoke to him. I never asked.

Still, there remained the experiment that I had wanted to do with extracellular gametes. I now found myself in Bob’s bailiwick working together with Dr David Chen, recently hired by Bob as his associate. Together, we conducted the experiment. I made semi-purified preparations of extracellular gametes of *P. gallinaceum*, X-irradiated them to inactivate any live asexual parasites and injected them intravenously into the wing veins of chickens at three weekly intervals. A month after the last injection, the birds were given blood infections of *P. gallinaceum*.

Virtually normal parasitaemias, asexual and gametocytes, male and female, followed. Daily through the normal period of their infectivity, I fed mosquitoes directly upon the birds. They failed, almost completely, to produce oocysts in the mosquitoes (the reduction in oocyst numbers exceeded 99% relative to the unimmunized control).^13^

When I tested sera from the immune birds, they powerfully agglutinated male gametes, as had those in Bob’s experiments, though I did not report on this particular fact until the publication of my next set of investigations.

These began with a comparative test of immunization with gametocytes versus extracellular gametes. The results appeared to show that the semi-purified extracellular gamete preparations were, indeed, more effective in inducing immunity which suppresses infectivity to mosquitoes than was whole blood containing gametocytes. At the time, it was a satisfying result. I had, after all, put the argument that the extracellular gametes would have cell surface antigens that the gametocytes did not. This, however, as I learnt from later work, is not the case. The antigens that are on the surface of extracellular gametes in a mosquito midgut following a blood meal, are already present in/on the mature intracellular gametocytes as they circulate in the blood. But there is a, actually rather extraordinary, twist to this matter. There is one antigen on the surface of the gametes that is, indeed, subtly, and crucially, different from its form in the gametocytes.

Antibodies by a ‘gamete agglutination’ test (that I conducted routinely and very manageably on a large scale using gamete preparations made courtesy of SA medium and all that we had learnt of the in vitro controls of gametogenesis), continued in the blood circulation of gamete-immunized chickens for several months after the completion of an immunization. Thereafter, titers of the anti-gamete antibodies (it was an assumption, but a pretty reasonable one, that they were antibodies) tended to fade out completely.

However – and in spite of declining titers of anti-gamete antibodies – when immunized birds were given a blood infection of the malaria parasites, their infectivity to mosquitoes remained firmly suppressed. Indeed, this was found even when anti-gamete antibodies were undetectable at the time that a blood infection was initiated.^14^

Now listen up, because this next bit is very important. The undiminished suppression of their infectivity to mosquitoes was because in the immunized birds, blood-induced infections of *P. gallinaceum* were accompanied by the almost instantaneous return of gamete-immobilizing, infectivity-suppressing antibodies. I’ll even say that again. The birds had been immunized with gametes. The anti-gamete antibodies eventually ceased to be detectible. The immunized birds were then infected with *P. gallinaceum*. Suddenly there were high titers of anti-gamete antibodies and the *P. gallinaceum* infections were unable to infect mosquitoes.

Indeed, the lower the antibody titers were, including their total absence, at the time of being given a blood infection, the more meteoric was their rise. So rapid would be their return that, from the earliest time point in the blood infections, when parasites were almost undetectable in the blood by light microscopy, the infectivity of the birds to mosquitoes remained highly suppressed.

The interpretation was, and is, as follows. Once a bird has been effectively immunized with gametes of the malaria parasites, any subsequent blood infection of the parasites boosts the anti-gamete antibodies to such high levels that they, the immunized chickens, are mostly unable to infect mosquitoes.^14^ This implies that the relevant gamete antigens must be present in the blood circulation where they can, and do, boost any previously induced anti-gamete immunity.

We now know – as just emphasized above – that these antigens are, indeed, present in the gametocytes themselves. It is the reason that Clay Huff and Bob had got their results.

If all this were to apply in humans in malaria-endemic areas, then a gamete-vaccinated population should remain permanently unable to infect mosquitoes with malaria. This is because, should, at any time, a vaccinated individual become infected with malaria, the blood infection would instantly boost their anti-gamete immunity back to infectivity-suppressing levels.

## BACK ON THE ROAD

A new post-doctoral scientist, Joan Rener, had meanwhile arrived in the *Malaria Section* and joined me on the gamete work. Initially, Rener set about making mouse monoclonals against gametes of *P. gallinaceum* while I turned out the preparations of gametes for immunizing them. I tested the mAbs for their effects on the infectivity of *P. gallinaceum* gametocytes to mosquitoes by membrane feeding. And I examined under a microscope, the effects of the same mAbs upon extracellular gametes.^15^

We did, indeed, find mAbs that blocked infectivity to mosquitoes. It was a curious situation, however. Though all of the mAbs that we tested in membrane feeding had been selected because they reacted with the gametes in agglutination tests, none of them, by themselves, had much effect upon infectivity of *P. gallinaceum* gametocytes to mosquitoes in a membrane feeder.

Two of these mAbs, however, when mixed together, blocked infectivity fairly strongly (around 85% overall). Separately, each mAb lightly agglutinated male gametes, leaving them free to wriggle vigorously. When mixed together, however, they caused a gruesome type of male gamete agglutination that I called ‘rope-like’ agglutination. The gametes became stuck together and almost totally immobilized in rope-like bundles in which they squirmed feebly. It was a striking, synergistic reaction between the two mAbs.^15^

In due course, more mAbs were produced against gametes of *P. gallinaceum*. Few had much effect by themselves; but, in pairwise combinations, several suppressed infectivity by 90–98%.

Then I changed the membrane feeding protocol. In some kind of “purist” mind-set (I suppose to test the effects of the antibodies themselves without any other agents involved), the membrane feedings had always been done using heat-inactivated serum as the carrying medium for the gametocytes and mAbs (typically at a dilution of one to four or so in the serum). Heat inactivation destroys *complement*. And Complement, in the right circumstances, destroys alien cells, in effect by exploding them. It is a very lethal (to any cell that does not belong to one’s own body) aspect of an immune response. I now decided to use freshly drawn human serum, without heat inactivation and with its complement system still intact, as the diluent for some experiments.

The effect was dramatic. When mAb combinations that suppressed by 90 to 98% in heat-inactivated serum were tested in serum which had its complement system intact, the infectivity of the gametocytes was suppressed by 100%. It was total wipeout – without exception. The full, and totally lethal to the sexual parasites, effects of the mAbs were all complement dependent! This had, and has, significant implications for what exactly are the mechanisms of transmission blocking immunity.

All this, interesting, and I am sure important, as it is, is given here as context for an observation that Rosenberg made concerning the zygotes of *P. gallinaceum*. I was intrigued by it and I asked his permission to investigate it. Permission was given. The observation was this.

Rosenberg had noticed that, whereas zygotes from the preparations I had shown him how to make survived happily in the presence on non-heat-inactivated chicken serum, the zygotes were lysed if placed in non-heat-inactivated human serum.

Together with Cyndi Grotendorst, I set out to investigate this peculiar – as it seemed to me at the time – and also puzzling phenomenon. It was puzzling because I had recently conducted the studies described previously in which *P. gallinaceum* gametocyte-infected blood had been fed to mosquitoes in the presence of non-heat-inactivated (I shall henceforth call this ‘native’) human serum. And in this work, there had been no reduction in the infectivity of the gametocytes in the presence of native human serum – which there certainly would have been if the zygotes were being lysed in its presence.

How could gametocytes happily infect mosquitoes in the presence of native human serum if this same native serum lysed the zygotes as soon as they formed (a matter of minutes later) into a mosquito midgut? And what was it, anyway, about human serum that so dramatically exploded *P. gallinaceum* zygotes?

What transpired was as follows.

Not only native human serum, but native serum from most of the species of animal we tested – duck, sheep, guinea pig, pig, rhesus monkey – lysed *P. gallinaceum* zygotes to one degree or another. Only native turkey serum had no effect upon the zygotes, and turkeys, of course, are closely related to chickens. Curiously, rabbit serum was also fairly harmless to the zygotes. Moreover, and as we now fully expected, zygotes of *P. gallinaceum* almost completely failed to infect mosquitoes in the presence of native serum from two non-gallinaceous species (human or guinea pig). The same zygotes were, of course, highly infectious to mosquitoes when fed in the presence of native chicken serum.

Something, therefore, about chicken serum rendered it a completely safe environment for *P. gallinaceum* zygotes, while something about the sera from most of the other species was highly, or totally, lethal to them.

To cut the story of an intricate series of investigations short, that lethal something was found to be the so-called *Alternative Pathway of Complement* (APC).^16^

## THE TARGET ANTIGENS OF MALARIA TRANSMISSION BLOCKING IMMUNITY

For several years around this period, my daily task became making and supplying my colleagues, and sometimes myself, with preparations of *P. gallinaceum* gamete and zygote material as just described. Our ‘immunochemical’ objectives were first to characterise the protein antigens present on the gamete surfaces and then to determine which (they have always been assumed to be proteins – whether rightly or wrongly I still cannot say) were the actual targets of the *P. gallinaceum* transmission blocking mAbs described above.

Male and female gametes and newly fertilized zygotes shared three surface proteins, one of which appeared at a molecular size of about 240 kDa and the others of which were around 56 and 54 kD.^17^^,^^18^ Both immunologically and otherwise, these three proteins on the male and female gametes and zygotes were indistinguishable from each other. All three were always immunoprecipitated together by any of the anti-gamete *P. gallinaceum* transmission blocking mAbs except for two whose targets could not be identified (for technical immunochemical reasons).

The work to identify the targets of anti-gamete *P. gallinaceum* transmission blocking immunity never proceeded beyond this level of knowledge. It came to a full stop. This is because from the late 1970s it had become possible to conduct equivalent studies with the real thing – the human malaria parasite *Plasmodium falciparum*. There can now be no doubt that the three proteins of 240 kD, 56 kD and 54 kD identified on the surface of gametes and zygotes of *P. gallinaceum* are the equivalent of the Pfs230, Pfs48 and Pfs45 gamete surface proteins of *P. falciparum*. For reasons I will come to, these proteins, and above all Pfs230, are the molecules that remain the best candidates for an effective *P. falciparum* malaria transmission blocking vaccine.

However, before leaving *P. gallinaceum* as the pioneer system for exploring the possibilities for malaria transmission blocking vaccines, there is one more angle to talk about. It is the existence of post-fertilization targets for such immunity.

As I was about to find out through the *P. gallinaceum* work, there is a class of target antigens of malaria transmission blocking immunity that is made/expressed only inside a mosquito midgut. It is a protein of apparent size of around 26 kDa that comes to be expressed upon the surface of malarial zygotes four to five hours after fertilization, which is to say four to five hours after the ingestion of gametocytes by a mosquito in a blood meal.^19^^,^^20^

This 26-kD protein **is totally absent** in the blood stage parasites, although the mRNA to make it has been shown to be present in gametocytes. Its expression in the fertilized zygotes is an example of ‘post-translational’ control of protein expression. It – the 26-kDa protein – can be a target of transmission blocking antibodies. mAbs against this 26-kD zygote surface protein suppressed infectivity of *P. gallinaceum* gametocytes to mosquitoes by over 90%.^20^

## *PLASMODIUM FALCIPARUM* IS CULTURED IN THE LABORATORY, AND EVERYTHING OPENS UP FOR STUDIES ON GAMETOCYTES OF THIS HUMAN MALARIA PARASITE

Not long after I had begun to work with gametocytes of *P. gallinaceum*, one of the great openings for malaria research took place. This was the ability to culture the blood stages of *P. falciparum* in the laboratory.

This success is, or was for a long time, almost universally attributed to the laboratory of William Trager at the Rockefeller University in New York. Together with James Jensen in his laboratory, continuous growth of asexual blood stage parasites in culture in human red blood cells was reported in 1976.^21^ In the same year, however, David Haynes and colleagues^22^ at the WRAIR had independently reported similar success using a different system. I was immediately attracted to using these culture systems for studying gametocytes of *P. falciparum* and began culturing the parasites in the laboratory.

When I began to culture *P. falciparum* in 1976, it was with the primary objective of producing gametocytes that could infect mosquitoes. By now, I was already sold on the ideas of transmission blocking immunity and the biology of the sexual stages of malaria parasites generally.

Working now with Ray Beach, we spent weeks and months carefully nursing the cultures and observing the formation and maturation of the gametocytes, as would be described in Jensen’s paper. What we were looking for was evidence that the gametocytes were functional, of which the first indication would be showing that they could undergo gametogenesis and exflagellation. This had yet to be seen with *P. falciparum* gametocytes from culture.

And then one day – applying the pH 8 bicarbonate stimulation method we had worked out for gametocytes of *P. gallinaceum* – we saw it. Mature cultured gametocytes of *P. falciparum* were seen to exflagellate – real live wriggling male gametes breaking out of rounded up male gametocytes and wriggling off among the blood cells. A few days later, we were able to repeat the feat.

This was not the greatest intellectual achievement in the world. It did matter, though, and rather a lot. For it was a spell-breaker. After everyone working towards it, it felt that mosquito infection from cultures of *P. falciparum* might actually be possible. Before, it was something that gametocytes from *P. falciparum* culture might just not be capable of doing.

We wrote up the finding in short order and it was published in *Nature* without any difficulty at all^23^ (received 7th June, published 22nd July 1977, which, I think, shows the mood of the time). It was covered in the science column of the *Times* (of London) newspaper, the first and only occasion anything I have published has been so honoured in the general press.

This result was my only contribution to the eventual transmission of *P. falciparum* from culture to mosquitoes. The first actual success in this regard came from the laboratory of Jerry Vanderberg at *New York University*.^24^ This was followed shortly by the successful mosquito transmission of cultured gametocytes of *P. falciparum* by the workers at the *Catholic University of Nijmegan* in the department of Professor Jeup Meuwissen.^25^ This group rapidly became and remains, as far as I know, in a class of its own in its efficiency and capacity in infecting mosquitoes from gametocytes of *P. falciparum* grown in culture.

Set up in 1980 by Meuwissen for the specific purpose of working towards a *P. falciparum* transmission blocking vaccine, he had recruited to his laboratory Tivi Ponnudurai. He had been the youngest Professor ever appointed to the Chair in the *Department of Parasitology* at the *University of Colombo*. Now, in Meuwissen’s laboratory, Ponnudurai took on a role that was almost that of a glorified technician. Together with his superb assistant, Ton Lensen, they set about putting together a technology of automated culture of *P. falciparum* for the production of gametocytes for the infection of mosquitoes. It worked spectacularly and transcended anything that anyone else has aspired to, then or since. I will pick up upon this in a later section.

There were actually two other laboratories, both in London, that had succumbed to the curious fascination of malaria transmission blocking immunity. One was that of my old fellow PhD student of Geoffrey Beale in Edinburgh, Bob Sinden. Two years my senior, Bob was an electron microscopist by PhD training. He had worked on *Paramecium* for his PhD with Geoffrey and had then gone south to join Professor Garnham and his colleague at *Imperial College London,* Professor Elizabeth Canning, as their research assistant. Garnham was very interested in looking at malaria parasites by electron microscopy. Bob soon had matters going in this exercise at a pace driven by his own irrepressible enthusiasm for the sexual stages of malaria parasites.

Bob got hooked on watching the parasites exflagellate which, as we know, they have to do before a zygote/ookinete can be formed. And once he was hooked on exflagellation, nothing else satisfied. He did a study on exflagellation shortly before I got interested in it myself.^26^

And then came our demonstrations, Bob’s and my own, of anti-gamete malaria transmission blocking immunity. I have it from Bob’s (Sinden, that is) own lips, or pen, I forget which now – “Gamete power” he called it – and Bob Sinden was on board the transmission blocking immunity train. In the ensuing decades, Bob, soon to be Professor Sinden, has contributed a large literature and great moral and intellectual support to the field, and to the very notion of malaria transmission blocking immunity as a public health measure.

The other individual who took up the malaria transmission blocking immunity matter in this period was Kamini Mendis, then a PhD student with Professor Geoff Targett at the *London School of Hygiene and Tropical Medicine* (in Garnham’s old department). Her first paper on the subject came out in 1979.^27^ I have the story from her own lips. She was supposed to be studying anti-sporozoite immunity in the rodent malaria parasite, *P. yoelii killicki*, it – sporozoite immunity and vaccines against sporozoites – being all the rage.

Things weren’t going too excitingly when she saw and read Bob Gwadz’s (yes! Bob’s, not mypaper!) 1976 *Science* paper on anti-gamete transmission blocking immunity.^12^ Without telling her supervisor, Geoff Targett, what she was up to, Kamini did her own anti-gamete/gametocyte immunization experiments and got a fine transmission blocking result.^27^ From then onwards, Geoff, who was an immunologist who had worked on anti-malarial immunity over a number of years, switched from the conventional anti-asexual immunity and his intended anti-sporozoite immunity work, to study malaria transmission blocking immunity, and has done so ever since.

Kamini graduated with her PhD and returned to Sri Lanka to the *Department of Parasitology* at the *University of Colombo*, from which Tivi Ponnuduri, who was the Professor when she had come to London, had just left to go to Nijmegen.

## VACCINE RACES

Together with Jack Williams and Tom Burkot at the WRAIR – who were at this time being more successful than ourselves at infecting mosquitoes with *P. falciparum* gametocytes – and using the immunochemical methods that we had learnt in the equivalent *P. gallinaceum* work, we identified the target antigens of effective *P. falciparum* transmission blocking mAbs. They came out as two proteins on the surface of the *P. falciparum* gametes that we labeled Pfs230 and Pfs48/45. I now thought that the transmission blocking immunity field was poised to shoot forward ahead of any other malaria vaccine type. After all, we knew the target antigens for sure, and we knew mechanisms by which the immunity destroyed the parasites.

For other conjectured types of malaria vaccine, including sporozoite, the target antigens and immune mechanisms were really not known at all. Whatever anyone might have said, or now say, about this, it was guesswork . . . plausible scenarios extrapolated from the experiment. How then could they know – I mean know for sure – what antigen gene to look for?

We, of transmission blocking immunity, on the other hand, had only to identify and isolate the genes for these target antigens (Pfs230, Pfs48/45 and Pfs25, known with certainty courtesy of membrane feeding, monoclonal antibodies and immunochemistry), express them in recombinant form (i.e., re-inserted into a bacterium or other microorganism) in culture, and we would be on the way to having an actual malaria transmission blocking vaccine.

How wrong we were. Wrong, not in our knowledge of the target antigens themselves of transmission blocking immunity, but in our anticipation of a smooth sail through the technologies of the time towards an actual malaria transmission blocking vaccine. And once again, I am going to spare you the details. It would be almost another decade before the genes for all three of these proteins (Pfs230-, Pfs48/45- and Pfs25-type proteins) had been identified and sequenced. It would take another decade to express any of them – actually only one type, the Pfs25 and also the *P. vivax* equivalent, Pvs25 – in remotely immunogenic form. The Pfs and Pvs 48/45 and 230 have now moved promisingly forward through heroic and Herculean efforts in certain laboratories around the world. But here we are today, in 2018, still waiting for anything that could enter human field trials as an experimental malaria transmission blocking vaccine.

That was a lightning fast forward across 30-plus years of studies of malaria transmission blocking immunity and of aspirations for a malaria transmission blocking vaccine.

You might be wondering, why does it matter who gets there first? Silly question, and I have nothing to say, except that in the early 1980s none of us had an inkling of just how long and difficult a matter the making a vaccine, any vaccine, against malaria, would actually become. That said, I do believe that the studies showed something – that human sera from a malaria-endemic population can have *P. falciparum* gamete-specific antibodies in them. I’m not going to justify this statement for the reasons just given. But they left me virtually convinced that this is so, as subsequent studies have validated. Thus, the expedition was not a loss from the transmission blocking immunity point of view. It laid a foundation of confidence for later studies on natural, or endemic, malaria transmission blocking anti-gamete immunity.

The implication was that, once induced, the immune memory for transmission blocking antibodies was so strong that it would be summoned back into full and comprehensively effective force, just as soon as any malaria parasite against which it was directed came into existence again in the blood circulation. Such an immunized individual would never again transmit malaria. A population so immunized would never again transmit malaria no matter when or how it, malaria, might attempt to return. From whatever source infections might be introduced back into that population, there would never again be onward transmission from members of the population that had been transmission blocking immunized. Transmission blocking antibodies was so strong that it [*sic*] would be summoned back into full and comprehensively effective force, just as soon as any malaria parasite against which it was directed came into existence again in children or adults.

It was, and is, an extraordinary vision. Too extraordinary for almost anyone else I have ever known to consider or seriously listen to me about. And, of course, it was actually just one chicken that was tested and it was only six months after the last gamete immunization. Not exactly from immunization to eternity . . . but why not? One chicken and six months was only the distance the observations had gone so far.

The intensity of malaria infection rate as experienced by a human population varies hugely. Under stable endemic malaria, transmission intensity varies over several orders of magnitude, from annual malaria inoculation rates approaching 1,000 per person per year, down to one or two.

In striking contrast, the reverse force of infection from the human population to the vector mosquitoes is extraordinarily invariable at every level of transmission, from the unbelievably high almost down to the point at which malaria transmission will suddenly cease (yes, I am proclaiming this and have not, for the sake of brevity, troubled to back this with data or argument – I could do so. And others should get out there and make some more measurements according to the method of Saul, Graves & Kay, 1990^28^). And that *reverse force of infection* into the mosquitoes hovers always at a nightly chance of around 1 in 10 human blood meals being infectious to a competent vector mosquito.

Put in other terms, wherever malaria is endemic, and regardless of the intensity of its transmission, from the perspective of a mosquito malaria vector, the size of the **Human Reservoir of Malaria** is mostly about the same – it is generally to be encountered in around one in 10 human blood meals.

## A SUMMING UP

As it multiplies in the blood of its human host, *P. falciparum* is constantly appraising and responding to its circumstances – ultimately to its best advantage for onward propagation through mosquitoes. Its calculations and responses concern phenomena of which gametocyte production itself is only one. For example, parasite antigen variation and the interaction of the parasites with the host immune system determine whether, and for how long, the asexual parasites survive in a human host. One might think of the asexual stages in their mouse-and-cat struggles with the host, as archers looking for the best moment(s) to release their arrows – the gametocytes.

Within the same blood infection, different lines of *P. falciparum* can, for reasons unknown, be hugely different in their capacities to make gametocytes. All, however, are subject to the variable circumstances that affect when and how many and what sort of gametocytes are made. And those variable circumstances have their influence always at the same particular parasite stage. Thus, all the merozoites from a single schizont have, during its growth in a red blood cell, been irreversibly imprinted with the instructions to form one particular type, and one type only, of blood parasite in the red blood cell that each will invade. Those merozoites from each individual blood stage schizont, must become either i) all male gametocytes, ii) all female gametocytes or iii) all asexual parasites.

No more questions about ‘What shall we be?’ can be asked or answered beyond this point, for the die has been cast.

## THE TARGET ANTIGENS OF NATURAL *P. FALCIPARUM* MALARIA TRANSMISSION BLOCKING ANTIBODIES

Using sera from Papua New Guinea, we were able to ask if there was any correlation between the amounts of antibody to certain specific *P. falciparum* gamete surface antigens and the effects of the sera on the infectivity of the parasites to mosquitoes.

And what we found was a very strong correlation between suppression of infectivity of *P. falciparum* gametocytes to mosquitoes and the amount of antibody in a serum to antigens on the surface of *P. falciparum* gametes. In particular, there was a strong correlation between antibody to Pfs230 on the gamete surface and the degree of suppression of infectivity of *P. falciparum* gametocytes to mosquitoes. The greater the amount of gamete surface Pfs230-specific antibody in a serum, the lower, on average, was the infectivity of *P. falciparum* gametocytes fed in its presence to mosquitoes. A weaker, and not significant, correlation was found with gamete surface Pfs48/45. The amounts of antibody against the internal – i.e., not on the gametes’ surface – proteins had little or no relationship to the effect of the sera on infectivity of *P. falciparum* gametocytes to mosquitoes.

Interestingly, residents of the malaria-free Highlands of Papua New Guinea (who had acquired their – possibly first – infection on a visit to the malaria-endemic Lowlands) were quite as likely as Lowlanders to have strong transmission blocking sera, and high levels of anti Pfs230 antibodies. And likewise, sera from both groups were equally likely to have no detectible antibodies to gamete surface Pfs230.

Please note that such ‘instant’ acquisition of an effective transmission blocking immunity by malaria-naïve individuals (from the Highlands) contrasts dramatically with the long-established generalisation that protective immunity against the disease-causing blood stages of malaria parasites takes years of continuous exposure to malaria to acquire. Curious and interesting. But here is something equally so.

If you look at our Papua New Guinea data,^29^^,^^30^ you will find that the amount of anti-Pfs230 antibody in any serum was either very low or absent (in about 27 of the sera), or it was very high, 10 to 20 times higher than a typical “very low” value (in about 13 of the sera). Only in a small number (about eight) was the amount of anti-Pfs230 antibody intermediate. By contrast, almost all the sera in our study contained moderate to high levels of antibodies to the internal gamete/gametocyte antigen, Pfs27.^28^ And this – a rather even response across all sera – applied generally to the antibody responses to other internal gametocyte antigens.^7^

Thus, while there was a comparatively even antibody response to the internal antigens of gametocytes/gametes of *P. falciparum* among the Papua New Guinea sera, the antibody response to the gamete surface antigen, Pfs230, was mostly either full on or full off. And this was regardless of the degree of previous exposure to infection with *P. falciparum*.

I end with this question left hanging . . . why were about half of those who had clearly been exposed to *P. falciparum* gametocyte antigens and who had made lots of antibodies against their internal antigens not making antibodies to gamete surface Pfs230?

## BACK AGAIN TO REAL-LIFE MALARIA TRANSMISSION

And so, I come to some further studies that were done on the effects of complement and malaria-endemic human sera on the infectivity of *P. falciparum* to mosquitoes. These were conducted in Edinburgh by Julie Healer under the primary supervision of Eleanor Riley. Julie was set the task of investigating the role of Complement in anti-*P. falciparum* gamete immunity in human sera from The Gambia in West Africa.

In the first part of these studies,^31^ Julie focused upon in vitro complement-dependent lysis of female gametes of *P. falciparum* and its relationship to the specificities of antibodies in the Gambian sera to antigens of sexual stages of *P. falciparum*.

And what Julie found was that Complement-mediated lysis of *P. falciparum* gametes by the sera was strongly associated with antibodies to Pfs230. There was no association with antibodies to Pfs48/45 nor with antibodies to the intracellular antigen of gametes and gametocytes of *P. falciparum*, Pfs27/25. Interestingly, but fully to be expected, nor was there any association between serum-mediated complement-dependent lysis of the gametes and the presence of antibodies to the ‘tail’ in the Pfs260 precursor of Pfs230. This ‘tail’, you will remember, is present on the gametes and gametocytes, so long as they are inside a host red blood cell (where antibodies cannot reach them). The ‘tail’ of Pfs230 is never present on the surface of the gametes of *P. falciparum* in a mosquito midgut where antibodies and Complement can and do reach them.

In a companion report, Julie tested the sera directly for their effects on infectivity (as opposed to in vitro gamete lysis) of *P. falciparum* to mosquitoes by membrane feeding.^32^ Though the numbers were too few to be statistically significant, the results indicated that complement-dependent suppression of infectivity was associated with the presence of anti-Pfs230 antibodies rather than with anti-Pfs48/45 antibodies. Enhancement of infectivity was associated with an absence of anti Pfs230 antibodies; it also had a small tendency to be associated with the presence of anti-Pfs48/45.

## THE LATEST NEWS.

Things have come a long way in the years since these studies were being completed. It is very exciting to see that Pfs230 is at last coming into play as a *P. falciparum* transmission blocking vaccine candidate.

And – what do you know? – the sera raised by immunization with the more effective of the Pfs230 vaccine constructs (I will discuss what exactly these are when I come to molecular structures and such of Pfs230 and it relatives) described in these reports reduce infectivity of *P. falciparum* to mosquitoes in membrane feeding assays by at least 80% and up to 100% if active Complement is present, but reduce infectivity only moderately (30–60%) if Complement is inactivated [. . .] just as anti-Pfs230 antibodies have always been found to do.

Transmission blocking vaccine development has continued involving the two other mosquito midgut-stage antigens I have mentioned so far, namely Ps48/45 and Ps25.

A recombinant construct representing Pfs48/45 of *P. falciparum* has induced antibodies that almost consistently reduce infectivity to mosquitoes by 99–100%; it is not clear whether complement was active or inactive in the tests.^33^ A construct representing the Pvs48/45 of *P. vivax* has induced antibodies that totally suppressed infectivity of *P. vivax* to mosquitoes in membrane feeding tests with active complement present. Unfortunately, no controls were included for the effect of the antibodies when complement is inactivated.^34^

Vaccine development with Ps25 has a long history. Its current state may be usefully represented by two articles on both Pfs25 of *P. falciparum* and Pvs25 of *P. vivax*.^35^^,^^36^ The effectiveness of the immunity induced by the vaccine constructs is a close function of the amount of anti-Ps25 antibody induced. The effect is always totally independent of active complement.^16^

## Pfs230, Pfs48/45 AND Pfs47 CARRY THE FERTILIZATION LIGANDS OF MALARIA PARASITES.

As we have seen, Pfs230 and Pfs48/45 are the predominant protein molecules on the surface of male gametes of malaria parasites. They are also among the predominant proteins on female gametes together with several other proteins. These proteins include the one that is now called Ps47, a molecule that had been identified on female gametes and zygotes in our early work with *P. gallinaceum*.

[*Editor’s note: “In addition to the importance of the Pfs genes in bringing the male and female gametes together, it was found in 2008 by O. Billker and W. J. Snell that a plant sterility gene HAP2 functions in membrane fusion in fertilization of *Chlamydomonas* and *Plasmodium* gametes.*^37^* This association of *Plasmodium* with a plant gene is not surprising in that *Plasmodium* comes, in part, from a plant.”* —*Louis H. Miller*]

## BIOINFORMATICS AND COMPUTER SIMULATION STRUCTURAL ANALYSIS OF THE Pfs230 FAMILY.

Around 2003, I met Dietlind Gerloff who, as by now did most other Edinburgh biologists of a molecular persuasion, worked in a just-completed, very modern building connected to the *Darwin Building* at *King’s Buildings* and called the *Swann Building* (named after Michael Swann, a famous Professor of Zoology at Edinburgh from the 1950s and 1960s). Dietlind is herself a structural molecular biologist and a complete whiz at her subject, especially in regard to bioinformatics and computer-based investigations. She became interested in the Pfs230 family and came up with some dramatic new insights.

First amongst these was the discovery that of all the proteins on Planet Earth then known to science, that which bore the greatest similarity to Pfs230 and its family was one from *Toxoplasma gondii* called SAG1.^38^

Now *T. gondii* belongs to the great family of organisms, protozoa indeed, called the *Apicomplexa*, to which malaria parasites themselves belong. All *Apicomplexans* are parasitic protozoa, which – being protozoa – is to say that they exist as single cells throughout their life cycles. All invade and grow inside cells of their host animals during most of their ‘asexual’ development. And all have an extracellular sexual phase which involves fertilization of male and female gametes of the parasites within the lumen of a host animal’s gut.

So Dietland’s finding, in an otherwise presumably unbiased computer screening of the planetary data bases, of a special similarity between the sequence of Pfs230 and that of a protein of another *Apicomplexan*, had an immediate ring of interesting plausibility about it.

With the assistance of her MSc student, Siarhei Maslau, Dietland proceeded to produce computer simulations of possible three-dimensional structures for double-domain units of the Pfs230 family. For this she used the known structure of the double-domain unit of SAG1 as a ‘framework’ from which to derive the computer simulations. SAG1 itself is actually a so-called ‘*homo dimer*’ consisting of two identical double-domain units in a non-covalently bound association showing computer-derived representations of the double-domain structure of SAG1.

The computer program used by Dietlind and Siarhei came up with thousands or tens, or hundreds, of thousands – I forget how many – possible three-dimensional structures for a Pfs230 family double domain. They were ranked in some kind of order of plausibility by the computer.

Amongst the top handful was one that was compatible with my predicted disulphide bonding models. It was too good not to be true and we chose it as our structural model prediction for the Pfs230 protein family domains.^38^ Seven years later, a MR spectroscopy structure of the Pf12 protein was published.^39^ It shows disulphide bonding for Pf12 exactly as originally predicted for this family of proteins^19^ and in Dietlind and Siarhei’s analysis.^38^

Looking at the pictures of 3D structure in this paper by Arredondo *et al.* 2012,^39^ and those of Dietlind’s computer simulations in Gerloff *et al.* 2005,^39^ it is not possible to know if Dietlind’s predicted twists and foldings are precisely correct or not. This is because Arredondo *et al.* 2012^39^ have analyzed only Pf12 (domain 2 actually) and Dietlind did not, to my knowledge, produce a computer model of Pf12. However, the models she did produce – for example of Pfs47 and Pfs230 domains 3 and 4 do follow closely, as near as one can see, the 3D structures of the ribbon diagrams of Arredondo *et al.* 2012.^39^ I have, however, a scientific criticism of one statement in this paper. It is in the introduction and is as follows ‘the 6-cysteine *Plasmodium* gamete-surface homology s48/45 domain *originally identified* by Williamson *et al*. . . .’

The statement is factually incorrect because the ‘6-cysteine *Plasmodium* gamete-surface homology s48/45 domain’ was not identified by Williamson *et al.* 1993.^40^ These authors explicitly described a ‘*seven cysteine* motif present six times in *P. falciparum*’ which they also noted to ‘exist as a single copy in Pfs12’. Without identifying and lining up the series of four distinct cysteine-containing motif types (I had identified five motif types, but one of these, Motif 5, never contained cysteines) as I had done, Williamson *et al.* 1993 did not – and could not have – detect(ed) the six cysteine-based pattern with its crucial structural significance. From the ‘seven cysteine’ motif no insight into the structures of these molecules was possible and none was offered.

Back now to 2004. With the results of Dietlind’s analysis, we now had an insight into what the Pfs230 family double domains might actually look like. And we had precise definitions and descriptions of the layout and interactions within the different parts of a Pfs230 family double domain unit Figure 2 from Gerloff *et al.* 2005^38^ demonstrating the structural equivalence between SAG1 and the Pfs230 family of proteins represented here by Pf12.

## THE CURIOUS POLYMORPHISMS OF Pfs230 AND ITS RELATIVES.

I will come shortly to the culmination of my speculations concerning these molecules. But first I must tell you about the polymorphisms within Pfs230, Pfs48/45 and Pfs47 and their distributions among *P. falciparum* populations from around the world.

Malaria parasites have many polymorphic genes and proteins about which many different stories can be, and have been, and will continue to be, told. Mine concern the gamete surface proteins Pfs230, Pfs48/45 and Pfs47.

I have already mentioned one polymorphism in these proteins. It is the polymorphism that determines whether mAbs against epitope region II of Pfs48/45 can, or cannot, suppress the infectivity of gametocytes of *P. falciparum* to mosquitoes.

Though we had no knowledge of the sequences of the Pfs48/45 gene or protein when this finding was made, we now know that this polymorphism in Pfs48/45 epitope region II is in domain II of this molecule, at amino acid position 254 from the N-terminal end of this protein (from studies on Pfs48/45 polymorphism and epitope analysis combined with the amino acid sequences of Pfs48/45 in isolates 3D7 and HB3 of *P. falciparum*).

Now, apart from the polymorphism just mentioned in epitope region II/domain II of Pfs48/45, no other polymorphism has (to my knowledge) ever been identified that has any effect upon the ability of an anti-gamete antibody to block malaria transmission. And this is remarkable because there are lots and lots of polymorphic amino acid positions in all three of the gamete surface proteins that I am concerned with here – namely Pfs230, Pfs48/45 and Pfs47, and in their equivalents in *P. vivax* – Pvs230, Pvs48/45 and Pvs47.

Now that’s all as may be. But here’s what I really want to point out. Strikingly different sets of polymorphisms in all of these proteins tend to collect in, and to distinguish between, malaria parasite populations from different geographic regions, including regions within a continent. This was first reported for polymorphisms of Pfs48/45.

And there must be a very special reason for the geographic clustering of these particular polymorphisms of Pfs48/45, because other genetic markers of other genes of *P. falciparum* don’t show the same clustering according to region.^41^

A comparable geographic clustering of polymorphisms of Pfs47 has also been reported^42^ and, although it has never been published, colleagues with whom I worked have also collected sequences indicating dramatic regional clustering of polymorphisms of Pfs47.

Strong regional bias in the distribution of their polymorphisms has been reported for all three *P. vivax* proteins – Pvs230, Pvs 48/45 and Pvs47.^43^^,^^44^ Only for Pfs230 of *P. falciparum* does the regional clustering of polymorphisms seem to be less pronounced, though the number of parasite isolates involved has been generally not large in this case.

Malaria transmission is sustained by distinct types and species of *Anopheles* mosquito in different geographic regions and it is well established that regional vectors are often less susceptible, or totally refractory, to malaria parasites from outside their own geographic region. Hence, regionally clustered polymorphisms in proteins involved in infecting mosquitoes – as malaria gamete surface proteins obviously are – might, indeed, be adaptations to infecting the regional mosquito vectors. The idea has a ring of plausibility.

And this is especially so since the recent finding by Alvaro Molina Cruz and colleagues at the *National Institutes of Health* that the Pfs47 molecule is involved in protecting *P. falciparum* from a Complement-like activity in the African mosquito and malaria vector, *Anopheles gambiae*.^45^ This being the case, it uncovers a second role for Pfs47 in addition to that as a female gamete fertilization ligand. It also fits with the continued presence of Pfs47 on the fertilized zygote through to mature ookinete in the midgut of a mosquito and the parasite’s exposure to mosquito immune systems.^19^

It has always been my temptation, nevertheless, to speculate whether anything interesting about the three gamete surface molecules, Pfs230, Pfs48/45 and Pfs47, has something to do with fertilization. So, I have long wondered if the polymorphisms in Pfs230, Pfs48/45 and Pfs47 might be at the business ends of the fertilization ligands and, in so wondering, if some combinations of polymorphisms in these proteins might make them more – or less – compatible as fertilization ligands.

Could this account for the regional exclusion of certain sets of polymorphisms and the selective inclusion of others? Could particular combinations of polymorphisms among these proteins work better for fertilization than do others, thereby creating geographic enclaves of sexually compatible *P. falciparum*? This, indeed, was the conjecture of David Conway and colleagues.^41^

I should say that this general idea is not without precedence. In ciliate protozoa there are things called ‘mating types’ (worked with by, amongst others, my old PhD supervisor Geoffrey Beale). The defining feature of ‘mating type’ is that some combinations of stocks of a particular species of ciliate, e.g., *Paramecium aurelia*, can mate together while other combinations can’t. And, as quoted by Conway *et al.* 2001,^41^ there have been reports on other organisms along similar lines.^46^^,^^47^

## ‘THE MEANING OF LIFE, THE UNIVERSE AND EVERYTHING!’.

I am – it may come as some relief – approaching the end of this particular tale . . . so far. When I thought that I had, indeed, reached its sudden conclusion – on 9th of July 2004 at 5.00pm – I impulsively wrote upon my notes ‘*The Meaning of Life, the Universe and Everything!*’ (a quote from the title of the book by Douglas Adams).

Now I have always been pleased by the relatively tidy organization of the connectivity figures (‘*squiggle*’ diagrams, as I always think of them) that I had used to represent the disulphide buttoning of Pfs230 and its relatives.^48^ One of the reasons that scientists jump out of bathtubs shouting ‘Eureka!’ when they discover, stumble upon, a scientific insight, is that a correct answer in Nature almost invariably has a visual and/or intellectual elegance to it. It is truly ‘beautiful’. Conversely, a wrong solution to a scientific enquiry usually has a distinct inelegance to it and is typically just plain ugly. With these thoughts in mind, I will now take you through the remains of this story.

Most of what I have written about so far in this Chapter I had been thinking about over many years. Aspects arising from them – my thoughts – were, from time to time, the subjects of Honours Student projects usually with an eye on a possible involvement of Pfs230, Pfs48/45 and Pfs47 in fertilization. Nothing conclusive ever came of it. Then one day – the 7th of July 2004, in the late afternoon – I decided to take the matter head on. I would play around with what I believed I knew of the structures of Pfs230, Pfs48/45 and Pfs47 until something did come of it. That is, I would scribble shapes and abstractions of these molecules on a piece of paper until something sensible, elegant . . . beautiful . . . fell into place before my eyes.

I began with a notion that the ‘*squiggles*’ could be simplified to the form of repeated flourishes as in the signature of Queen Elizabeth I . . . .

**Figure 1.**
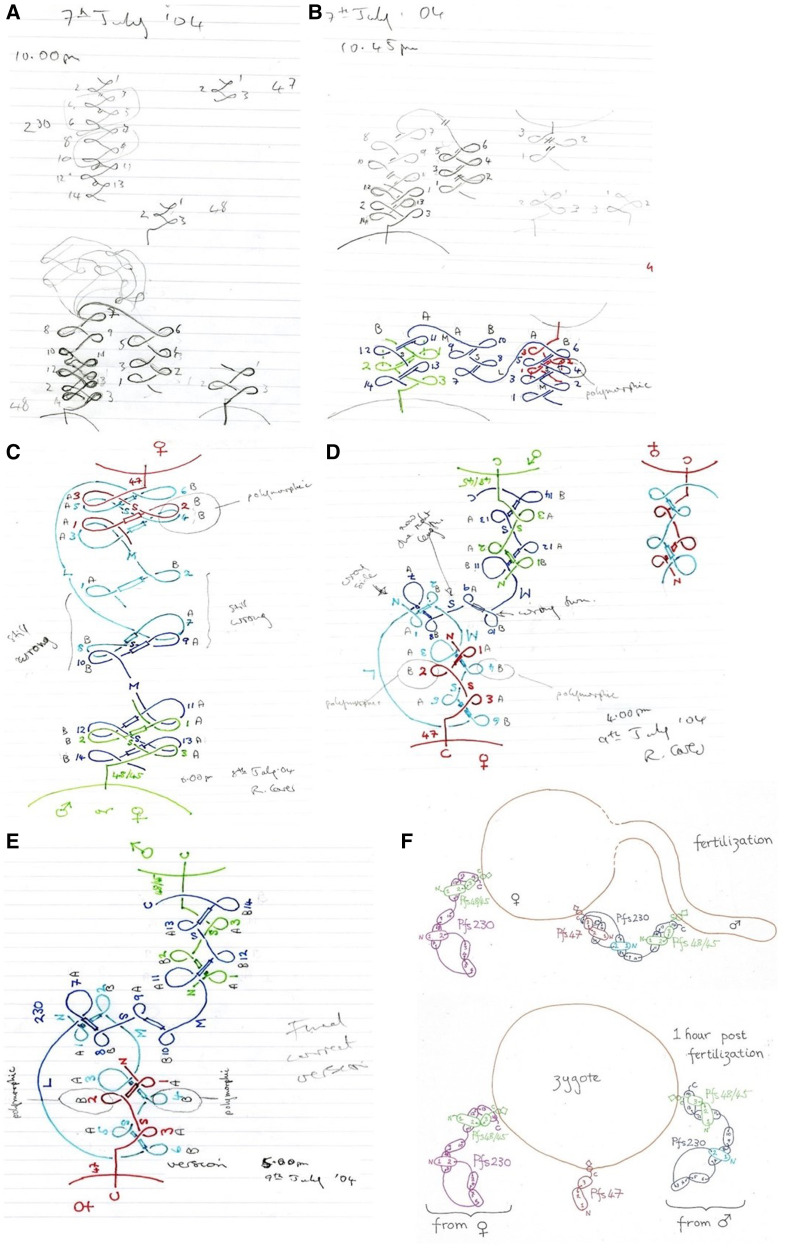
Five sheets of paper with my workings towards ‘*The Meaning of Life, the Universe and Everything!*’: (**A**) 10:00pm evening of 7th July 2004, (**B**) 10:45pm evening of 7th July 2004, (**C**) 8:00pm evening of 8th July 2004, (**D**) 4:00pm afternoon of 9th July 2004, (**E**) 5:00pm afternoon of 9th July 2004 – ‘*The Meaning of Life, the Universe and Everything!*’. (**F**) Model for interactions and behaviours of Pfs230, Pfs48/45 and Pfs47 in fertilization and post-fertilization in *Plasmodium falciparum*.

I was looking first to superimpose the ‘flourishes’ of Pfs230 (without the N-terminal glutamic repeat-rich tail) onto those of Pfs48/45, C terminus on C terminus – an easy task. This – the combination of Pfs230 with Pfs48/45 – would, in my mind, make up the male gamete fertilization ligand (Figure 1A). Next, I needed to join the free-floating (N-terminal) end of Pfs230 to Pfs47 on the surface membrane of the female gamete. I had to struggle a little to turn the N-terminus of Pfs230 on its head towards being able to link up with Pfs47. And I now realised that the superimposition of this N-terminal end of Pfs230 onto Pfs47 as it poked up like a tree from the surface of a female gamete was not satisfactory. It would result in an N terminus (Pfs230)-on-C terminus (Pfs47) arrangement and the opposite of the C terminus-on-C terminus rule which I had already intuitively set myself for the Pfs230 to Pfs48/45 interaction. Consistency in this matter, my instincts told, would be essential to any correct solution.

To achieve the C terminus-on-C terminus orientation for the Pfs230/Pfs47 interaction, I had to move things around. And now I had also made another decision, not strictly necessary – not yet anyway – for everything to work. It was to lay the N terminus of Pfs230 – not so that its first two domains lay next to the first two of Pfs47 (now C terminus on C terminus) – but so that domains 3 and 4 of Pfs230 did so.

I was beginning to think that I was getting somewhere and I carefully sketched out my ‘model’ in color – green for Pfs48/45, blue for Pfs230 and red for Pfs47. I thought it looked rather pretty (see my criterion for a successful scientific result, above). It was also showing some interesting features (Figure 1B).

One is that polymorphic domains of Pfs230 and Pfs47 (Pfs230 domain 4 and Pfs47 domain 2) were now juxtaposed (please note that in my workings and later, and as I will now continue, I have confusingly gone from using Roman numerals, e.g., IV and II, for domain numbering, as in the published literature, to Arabic, i.e., 4 and 2).

I do not remember, at this late time after the event, but I must have deliberately chosen the packing of Pfs230 with Pfs47 to achieve this very outcome. If so, it was soon to be vindicated by what was to come. Also of interest was that the arrangement left Pfs230 domains 7, 8, 9 and 10 free and unattached to anything else. These domains thus became a bridge between its attachment to Pfs48/45 on the male gamete and Pfs47 on the female; a rather inevitable outcome one could think. Less obvious, however, was that there would be a bit of Pfs230 – the N-terminal domains 1 and 2 – sticking out beyond where it ligated with Pfs47. I was not sure that I had quite got to the bottom of everything yet, but all in all, I liked what I had.

By now it was almost midnight and I was flying next day to Geneva to meet with Kamini Mendis, who was by now working for the ‘*Roll Back Malaria*’ program at the WHO (of which there are stories to be told elsewhere).

I remember playing with the problem on the flight and getting increasingly frustrated that it was not actually working. I recall, nevertheless, enthusiastically showing Kamini what I was up to and then working again on it that evening at her apartment in the Petite Sacconex neighborhood of Geneva (a 15-minute walk from WHO Headquarters).

That evening’s fiddling produced an attempt that didn’t look very pretty – for elegance, I would give it a B minus. It had, nevertheless, two elements that were essential towards the final outcome. It showed that I was trying to move the Pfs230 N-terminus domains 1 and 2 and the four middle domains, 7 to 10, together, and it has a first attempt towards intercalating these Pfs230 domains with each other (Figure 1C).

The next day, Friday 9th July 2004, produced the result that has stood the test of time and fits every piece of structural information that there is (up to now, 19th July 2015) and known to me. It was the impulse of the previous evening to join the N terminus of Pfs230 to its middle domains that did it. At 4.00pm on the evening of 9th July, I thought that I had, indeed, ‘done it’ (Figure 1D). Then I noticed a small error in the twisting of the Pfs230 (the Elizabethan loops were, and are, only a simplifying way of doing this investigation and I have not tried to think if the directions of the twists actually matter for correct structure). Anyway, for complete correctness in the terms of this analysis, I re-drew the image with the Pfs230 twist the correct way and marked it ‘5.00pm 9th July ’04; Final correct version’ (Figure 1E).

It is ‘*The Meaning of Life, Universe and Everything!*’ . . . if you are a malaria parasite.

And it shows, amongst anything else that you may take from it, something that had been gradually emerging from all the previous analyses as they came up. I had written ‘L’ or ‘M’ or ‘S’ next to linear sections joining the double domains. This is because, in my original connectivity diagrams, these sections – the ‘J loops’ – are either Long (L), Medium length (M) or Short (S). And the structure represented in ‘*The Meaning of Life, the Universe and Everything!*’ (Figure 1) accounts, in every instance, for the Long, Medium length and Short connections in all three molecules, Pfs230, Pfs48/45 and Pfs47.

Without the bonding of the N-terminal domains 1 and 2 of Pfs230 to its middle domains, 7 and 8, there would be no need to draw the long line marked ‘L’ – at least on paper. And I assumed, and am assuming, that if this section is long on paper, it is likely to be long in reality as well; likewise for medium, ‘M’, and short, ‘S’, connections between double domains.

Furthermore, without having had to tuck away the two N-terminal domains of Pfs230 in the way just described, there would not be a compelling reason for Pfs230 domains 3 and 4 to juxtapose with Pfs47 domains 1 and 2 respectively, and so require that the polymorphic domain 2 of Pfs47 lie opposite polymorphic domain 4 of Pfs230. As will be shown below, these two polymorphic faces look as though they should come very close to each other in the interaction between Pfs47 and Pfs230.

## THE ‘KISSING COUPLE’.

Here the matter lay for several years. Dietlind and I had hopes that there might be ways to test ‘*The Meaning of Life, Universe and Everything!*’ through her wizard computer analyses. She had actually begun to do so with her ‘space filling’ computer models when, alas, in late 2004, Dietlind left Edinburgh for a position in California. By myself there was little – nothing apparently – further that I could do.

Then, shortly before I retired from the University of Edinburgh in September 2010, I found myself once again playing with the images from ‘*The Meaning of Life, the Universe and Everything!*’. I had decided to take into account the three-dimensional structure of the SAG1 molecule that Dietlind’s work had revealed as representing the underlying structure for the Pfs230 family. And the thing about SAG1 is that it is actually two independent double-domain units that link (ligate) together as what I fancifully (as usual) called a ‘*Kissing Couple*’ for it brought to my mind the famous sculpture by August Rodin (actually sculptures because he/his workshop apparently produced rather a lot of copies of it) entitled ‘*The Kiss*’.

In the ‘*Kissing Couple*’ model, the double-domain unit of the Pfs230 family becomes a lozenge with N- and C-terminal ends. The ‘*Kissing Couple*’ itself becomes two lozenges facing each other in an X formation, N terminus opposite N terminus and C terminus opposite C terminus.

It shows (as was indicated in the original of 9th July 2004) the juxtaposition of the polymorphic regions of Pfs230 domain 4 and Pfs47 domain 2 (Figure 4 from Gerloff, D.L. *et al.* 2005^38^ showing the structural positions of the polymorphisms of domain IV of Pfs230). Why, and with what effect, these polymorphisms are just exactly there, right where the male and female fertilization ligands come into contact – who knows? But whatever else it achieves, the ‘*Kissing Couple*’ model allows visually easy-to-follow representations of all the facts and hypotheses concerning these molecules as presented in this and previous Chapters.

And there is why, and how, I have come to imagine Pfs230, Pfs48/45 and Pfs47 locked in the intimate embrace of the fertilization ligands of *P. falciparum* malaria (Figure 1E).

[*Editors’ Postscript: Richard’s intuition regarding both the structure of the six-cysteine domains of these proteins*^38^* and of the interactions between Pfs230 and Pfs^48^/45 during gamete fertilization,*^49^* were ultimately proved correct in later independent studies.*]
